# Effects of docosanyl ferulate, a constituent of *Withania somnifera*, on ethanol- and morphine-elicited conditioned place preference and ERK phosphorylation in the accumbens shell of CD1 mice

**DOI:** 10.1007/s00213-022-06069-w

**Published:** 2022-01-28

**Authors:** Riccardo Maccioni, Marcello Serra, Jacopo Marongiu, Filippo Cottiglia, Elias Maccioni, Valentina Bassareo, Micaela Morelli, Sanjay B. Kasture, Elio Acquas

**Affiliations:** 1 Department of Life and Environmental Sciences, University of Cagliari, Cagliari, Italy; 2 Department of Biomedical Sciences, University of Cagliari, Cagliari, Italy; 3 Center of Excellence for the Study of Neurobiology of Addiction, University of Cagliari, Cagliari, Italy; 4 Pinnacle Biomedical Research Institute, Bhopal, India

**Keywords:** Conditioned place preference, Docosanyl ferulate, Ethanol, ERK, Morphine, pERK, *Withania somnifera*

## Abstract

**Background:**

Docosanyl ferulate (DF) is a behaviourally active GABA_A_ receptor complex (GABA_A_R) agonist, recently isolated from the standardized methanolic extract of *Withania somnifera* Dunal (*WSE*) root. Previous studies have shown that *WSE* prevents both ethanol- and morphine-dependent acquisition and expression of conditioned place preference (CPP) and stimulation of dopamine release in the nucleus accumbens shell (AcbSh).

**Aims:**

The study aimed at determining (**a**) whether DF contributes to *WSE*’s ability to affect the acquisition and expression of ethanol- and morphine-elicited CPP and, given that phosphorylation of extracellular signal-regulated kinase (pERK) in the AcbSh is involved in associative learning and motivated behaviours, (**b**) whether *WSE* and DF may affect ethanol- and morphine-induced ERKs phosphorylation in the AcbSh.

**Methods:**

In adult male CD1 mice, DF’s effects on the acquisition and expression of ethanol- and morphine-elicited CPP were evaluated by a classical place conditioning paradigm, whereas the effects of *WSE* and DF on ethanol- and morphine-elicited pERK in the AcbSh were evaluated by immunohistochemistry.

**Results and conclusions:**

The study shows that DF, differently from *WSE*, affects only the acquisition but not the expression of ethanol- and morphine-induced CPP. Moreover, the study shows that both *WSE* and DF can prevent ethanol- and morphine-elicited pERK expression in the AcbSh. Overall, these results highlight subtle but critical differences for the role of GABA_A_Rs in the mechanism by which *WSE* affects these ethanol- and morphine-dependent behavioural and molecular/cellular responses and support the suggestion of *WSE* and DF for the control of different components of drug addiction.

## Introduction

Drug addiction is defined as the progressive loss of control over drug taking caused by repeated exposures to addictive drugs. This is the consequence of a series of neuroadaptations, occurring in selective neural circuits, which leads to the development of a chronic neuroadaptive disorder characterized by behavioural alterations in which compulsive drug-seeking and high risk of relapse play a critical role (Berke and Hyman 2000; Koob 2006; Koob and Le Moal 2001; Nestler 2001; Volkow and Morales 2015). Ethanol and morphine are two substances that are well-characterized to induce such neuroadaptations. Accordingly, ethanol is one of the most used and abused psychoactive substances worldwide, is a high-risk factor for several multi organ diseases (Axley et al. 2019; Rehm et al. 2017) and is responsible for the potential of alcoholic drinks to trigger their pathological consumption (Abrahao et al. 2017). Morphine is, instead, the lead compound prescribed for the treatment of multiple and diverse chronic painful conditions and although developing addiction is not an issue in this case, a high rate of dependence in those who take morphine chronically has been reported with debilitating side-effects such as constipation and respiratory depression (Benyamin et al. 2008). Notably, although the mechanism of action by which ethanol and morphine may elicit addiction is different, though not fully understood, these drugs share the ability to increase mesolimbic dopamine (DA) transmission (Di Chiara et al. 2004; Bassareo et al. 2019, 2021) and induce the phosphorylation and subsequent activation of the extracellular signal-regulated kinases (ERKs) (Ibba et al. 2009; Porru et al. 2020; Rosas et al. 2016; Spina et al. 2015; Valjent et al. 2004), two biochemical indexes critical for addiction-related behaviour in laboratory studies (Di Chiara 1999; Di Chiara et al. 2004).

The conditioned place preference (CPP) paradigm, widely used to study the rewarding properties of unconditioned stimuli (drugs, food, sex, etc.), is endowed with great translational impact due to its face, construct and predictive validity (Tzschentke 2007). The acquisition and the expression of CPP are two critical phases of this paradigm. In particular, the acquisition of place conditioning is grounded on associative learning (Di Chiara et al. 2004) and represents the phase in which the reinforcing properties of an unconditioned stimulus are transferred to the conditioned, otherwise neutral, stimulus; on the other hand, the expression of place conditioning represents the phase in which the reinforcing properties of the unconditioned stimulus that have been transferred to the conditioned one are recognized (recalling) by the animals that may hence emit a response toward that stimulus (positive side-preference shift: animals spend, in the environment associated with the unconditioned stimulus, longer time than before conditioning). Thus, in translational perspective, acquisition and expression of CPP, by modelling two distinct critical conditions of the clinical, naturalistic, setting of drug addiction allow to investigate, respectively, the phase in which subjects attribute drug’s reinforcing properties to the context (acquisition and/or maintenance of drug-taking) and that in which the conditioned stimulus becomes eventually capable of triggering relapse into drug-taking (expression, reinstatement). This, in turn, makes treatments that may prevent any of these critical phases of drug addiction highly desirable.

ERKs are part of the mitogen-activated protein kinase (MAPK) signalling cascade and play a central role in signal transduction, neuroplasticity and gene expression (Sweatt 2004) as well as in the behavioural changes underlying the reinforcing processes induced by drugs of abuse (Valjent et al. 2005). ERKs are highly expressed also in reward-related brain areas and their activation through phosphorylation (pERK) occurs after both acute (Acquas et al. 2007; Brami-Cherrier et al. 2006; Ibba et al. 2009; Rosas et al. 2016) or chronic (Berhow et al. 1996; Muller and Unterwald 2004) administration of drugs of abuse. Notably, both acquisition and expression of place conditioning have been strictly associated with the increased activation, by phosphorylation, of ERKs. In particular, ERKs phosphorylation has been shown to be increased, in key brain regions such as those of the mesolimbic system and the extended amygdala, during the acquisition and expression of place conditioning (Gerdjikov et al. 2004; Mazzucchelli et al. 2002; Porru et al. 2020; Salzmann et al. 2003; Valjent et al. 2000, 2001). Accordingly, several studies have demonstrated that the pharmacological inhibition of the mitogen activating ERK kinase (MEK), the kinase responsible for ERKs phosphorylation (Sweatt 2004), prevents pERK expression and CPP acquisition elicited by several drugs of abuse (Gerdjikov et al. 2004; Lu et al. 2006; Porru et al. 2020; Salzmann et al. 2003; Spina et al. 2010; Valjent et al. 2000, 2001), including ethanol (Rosas et al. 2017), its metabolite, acetaldehyde (Correa et al. 2012; Spina et al. 2010) and morphine (Lin et al. 2010; Spina et al. 2010). On a similar vein, place conditioning and self-administration studies have shown that increased pERK is strictly associated with the emission of the acquired response (CPP expression) (Mazzucchelli et al. 2002; Porru et al. 2020) and, consequently, prevention of ERKs phosphorylation during the post-conditioning test prevents the behavioural outcome (CPP expression) (Rosas et al. 2017; Porru et al. 2020). Thus, increased ERKs phosphorylation appears involved, although with some exceptions (Rosas et al. 2018), not only in the attribution of motivational value to neutral stimuli when paired with the primary effects of addictive substances (acquisition of conditioned responses) (Gerdjikov et al. 2004; Rosas et al. 2018; Valjent et al. 2001) but also in the recognition and recall of drug-conditioned stimuli (expression, i.e. behavioural response to presentation of conditioned stimuli) (Mazzucchelli et al. 2002; Porru et al. 2020).

*Withania somnifera* (*WS*) Dunal is a medicinal plant originally included in the Ayurveda, the Indian traditional system of medicine, whose central properties extend from anxiolytic and neuroprotective to anti-inflammatory and anti-neurodegenerative (Dar et al. 2015; Maccioni et al. 2018; Singh et al. 2011). Interestingly, *WS*’s standardized methanolic root extract (*WSE*) has also been reported, in preclinical rodent models of drug addiction, to prevent the acquisition and the expression of ethanol (Spina et al. 2015)- and morphine (Ruiu et al. 2013)-elicited CPP. Moreover, recent evidence demonstrated that *WSE*, through a GABA_A_ receptor complex (GABA_A_R)-mediated mechanism, also prevents the ethanol- and morphine-dependent increases of DA transmission in the rat nucleus accumbens shell (AcbSh) (Bassareo et al. 2019). This evidence, given the role of mesolimbic DA in drug-elicited place conditioning (Acquas and Di Chiara 1994; Di Chiara et al. 2004; Fenu et al. 2006), suggests a plausible mechanism for *WSE*’s positive effects on motivated behaviours.

Based on these premises, given (**i**) the profile of docosanyl ferulate (DF) as most active constituent of *WSE* on GABA_A_R (Sonar et al. 2019) with anxiolytic properties without sedative, amnesic, motivational and motor coordination-impairing effects (Maccioni et al. 2021), and (**ii**) the importance of the proposed GABA_A_R-mediated mechanism for *WSE*’s actions on mesolimbic DA function (Bassareo et al. 2019), the present study aimed at verifying, whether DF could affect the acquisition and/or the expression of ethanol- and morphine-induced CPP. Moreover, based on the critical connection between CPP and ERKs activation (Gerdjikov et al. 2004; Lu et al. 2006; Mazzucchelli et al. 2002; Porru et al. 2020; Salzmann et al. 2003; Spina et al. 2010; Valjent et al. 2000; Valjent et al. 2001), the study also aimed at verifying whether *WSE*, at a dose (50 mg/kg) at which it prevents acquisition and expression of ethanol (Spina et al. 2015)- and morphine (Ruiu et al. 2013)-elicited CPP, and DF, at a dose (2 mg/kg), at which it shows robust anxiolytic properties with no undesired side-effects (Maccioni et al. 2021), could prevent pERK expression elicited by ethanol (Porru et al. 2020; Rosas et al. 2017) or morphine (Rosas et al. 2016) in the AcbSh.

## Materials and methods

### Animals

Adult male CD1 mice (22–24 g, Charles River, Calco, Italy) (*n* = 305) were housed in groups of eight per cage for at least 6 days before the experiments began, under a 12:00/12:00 h light/dark cycle (lights on at 08:00 a.m.) with food (Mucedola Srl, Settimo Milanese, Milan, Italy) and water available ad libitum. All the experiments were carried out during the light phase, between 09:00 and 18:00 h. The total number of mice used for CPP and immunohistochemistry experiments was 179 and 126, respectively. All the experimental procedures were performed in accordance with the principles of laboratory animal care, with the guidelines and protocols approved by the European Union (2010/63/UE L 276 20/10/2010) and with the approval of the local committee (authorization number 371/2020-PR). Every possible effort was made to minimize animal pain and discomfort and to reduce the number of experimental subjects.

### Drugs

Ethanol (EtOH) (Sigma-Aldrich, Milan, Italy) was diluted in saline (NaCl 0.9% w/v) to 20% (v/v) and administered at the dose of 2 g/kg (12.5 ml/kg volume injection). Morphine hydrochloride (MOR) (Franchini Prodotti Chimici Srl, Mozzate, Como, Italy) was dissolved in saline (10 ml/kg volume injection) and administered at the dose of 5 mg/kg. The standardized methanolic extract of the root of *Withania somnifera*, *WSE* (Natural Remedies Pvt Ltd, Bangalore, India) was dissolved in saline and administered at the dose of 50 mg/kg (10 ml/kg volume injection). Docosanyl ferulate (DF), synthesized (purity > 98% by HPLC) according to Sonar et al. (2019), dissolved in Tween 80 (Sigma-Aldrich, Milan, Italy) and suspended in saline, was administered at the dose of 2 mg/kg (10 ml/kg volume injection). Sodium pentobarbital (Pentothal Sodium, MSD Animal Health S.r.l, Italy) was dissolved in saline and administered at the dose of 50 mg/kg. All drugs were administered intraperitoneally (i.p.) at doses in accordance with previous experiments (Ibba et al. 2009; Maccioni et al. 2021; Porru et al. 2020; Rosas et al. 2016; Ruiu et al. 2013; Spina et al. 2015).

### Conditioned place preference (CPP)

The apparatus consisted of two rectangular Plexiglas boxes (48 L × 20 W × 30 H cm) separated by a guillotine door, placed in a sound-proof room with a constant light of 37.5 Lux (ELD 9010 Luxmeter, Eldes Instruments, Italy) provided by a 40 W lamp placed above each compartment. Different visual and tactile cues distinguished the two compartments: vertically striped black and white walls and white smooth floor for one compartment (A), and horizontally striped black and grey walls and fine grid floor for the other compartment (B). The spontaneous preference was randomly distributed between compartments (49% for compartment A and 51% for compartment B) and did not differ statistically amongst the experimental groups (Table 1).

**Table 1:** Average pre-conditioning test time (sec/900 ± SEM) (spontaneous preference) of the experimental groups of the acquisition and expression experiments

Experimental group	Spontaneous preference (seconds/900 ± SEM)	*N*	One-way ANOVA
Acquisition of ethanol-induced CPP			[*F*_(3,52)_ = 0.61, *p* > 0.05]
Veh/Veh	379 ± 15	12	
DF/Veh	348 ± 28	10	
Veh/EtOH	379 ± 24	16	
DF/EtOH	389 ± 16	18	
Acquisition of morphine-induced CPP			[*F*_(3,40)_ = 0.08, *p* > 0.05]
Veh/Veh	394 ± 16	12	
DF/Veh	386 ± 20	10	
Veh/MOR	382 ± 20	12	
DF/MOR	385 ± 18	10	
Expression of ethanol-induced CPP			[*F*_(3,35)_ = 0.16, *p* > 0.05]
Veh/Veh + Veh	394 ± 20	10	
Veh/Veh + DF	390 ± 18	8	
Veh/EtOH + Veh	376 ± 27	8	
Veh/EtOH + DF	387 ± 10	13	
Expression of morphine-induced CPP			[*F*_(3,36)_ = 0.03, *p* > 0.05]
Veh/Veh + Veh	394 ± 20	10	
Veh/Veh + DF	391 ± 18	8	
Veh/MOR + Veh	392 ± 16	9	
Veh/MOR + DF	387 ± 17	13	

#### CPP acquisition experiments

The experiment consisted of three phases. During the first phase (pre-conditioning test, day 1), the guillotine door was kept raised and each mouse was placed randomly in one compartment and given access to both compartments of the apparatus for 15 min (900 s). The time spent in one compartment was recorded and taken as indication of spontaneous preference. During the second phase (conditioning, days 2–5), mice of the experimental groups (as indicated above) were administered (pre-treatment) either vehicle (Veh) or DF and returned to their home cages for 30 min. At the end of this period, mice were administered (treatment) either vehicle (Veh) or ethanol (EtOH) or morphine (MOR) and exposed for 30 min to the given compartment. On the same day, 8 h later, mice of all groups were administered Veh or DF (pre-treatment) and, after 30 min, immediately after being administered the drug (EtOH or MOR) or Veh (treatment), were exposed to the opposite compartment for 30 min. The sequence of the administrations of Veh or DF, as pre-treatment, and of Veh or drug (EtOH or MOR), as treatment, was alternated in the following days so that on consecutive days mice never received Veh or DF (pre-treatment) and Veh or EtOH or MOR (treatment) administrations in the same order. During the third phase (post-conditioning test, day 6), 24 h after the last conditioning session, the guillotine door was kept raised and the time spent, out of 15 min, by each mouse in the drug-paired compartment was recorded. The conditions of the post-conditioning test were identical to those of the pre-conditioning test. Performances at the pre- and post-conditioning tests were videotaped and subsequently analysed in blind. A statistically significant difference between the time spent during pre- and post-conditioning tests (side preference shift) of the drug group with respect to that of the vehicle group was taken as indication of the development of place conditioning.

#### CPP expression experiments

The general protocol was the same of the one used for the acquisition experiments with two differences: (i) during conditioning (phase 2), mice were administered only Veh (pre-treatment) and either Veh or EtOH or MOR (treatment) (groups: Veh/Veh and Veh/EtOh or Veh/MOR) and (ii) 30 min before performing the post-conditioning test (phase 3), mice were administered either Veh or DF (groups: Veh/Veh + Veh, Veh/Veh + DF, Veh/EtOh or Veh/MOR + Veh and Veh/EtOh or Veh/MOR + DF). As for the post-conditioning test of the acquisition experiments, a statistically significant difference between the time spent during pre- and post-conditioning tests (side preference shift) of the drug group with respect to that of the vehicle group was taken as indication of the expression of place conditioning.

### Immunohistochemistry

Drug-elicited ERKs phosphorylation in the AcbSh is critical for the acquisition of drug-elicited place conditioning (Gerdjikov et al. 2004; Salzmann et al. 2003). The immunohistochemistry experiments of this study have been planned in order to investigate whether *WSE* and DF may prevent the ability of ethanol and morphine to elicit ERKs phosphorylation in the AcbSh. This was done in order to allow us to indirectly infer that *WSE*’s (Spina et al. 2015; Ruiu et al. 2013) and DF’s (present study) property to prevent CPP acquisition may be attributed to their ability to affect ethanol- or morphine-elicited ERKs phosphorylation. Thus, since for this technique, animals have to be sacrificed in order to allow processing their brains, distinct cohorts of animals were utilized for these experiments. Mice of different experimental groups were carried out in the experimental room and given 1 h of habituation time. Subsequently, they were administered Veh or *WSE* (50 mg/kg) or DF (2 mg/kg) (pre-treatment). After 30 min, mice were administered Veh or EtOH (2 g/kg) or MOR (5 mg/kg) (treatment). Experimental groups consisted, accordingly, in Veh/Veh (*n* = 8), *WSE*/Veh (*n* = 8), Veh/EtOH (*n* = 6) and *WSE*/EtOH (*n* = 6) and Veh/Veh (*n* = 8), *WSE*/Veh (*n* = 8), Veh/MOR (*n* = 8), *WSE*/MOR (*n* = 10) for the experiments performed with vehicle and *WSE* as pre-treatment; Veh/Veh (*n* = 8), DF/Veh (*n* = 7), Veh/EtOH (*n* = 7), DF/EtOH (*n* = 9) and Veh/Veh (*n* = 8), DF/Veh (*n* = 7), Veh/MOR (*n* = 8), DF/MOR (*n* = 10) for the experiments performed with vehicle or DF as pre-treatment. Mice of the ethanol-related experiments were anesthetized, with sodium pentobarbital (50 mg/kg), 15 min after the treatment (Ibba et al. 2009; Rosas et al. 2017), whilst subjects of the morphine-related experiments were anesthetized, with sodium pentobarbital (50 mg/kg), 20 min after the treatment (Rosas et al. 2016). Under deep anaesthesia, animals were subjected to transcardial perfusion with 0.9% NaCl followed by ice-cold 4% paraformaldehyde (PFA) in 0.1 M phosphate buffer solution (PBS) (137 mM NaCl, 2.7 mM KCl, 10 mM Na_2_HPO_4_, 2 mM KH_2_PO_4_, pH 7.4). After perfusion, brains were removed and post-fixed for 2 h in 4% PFA (4 °C). Two coronal brain slices (40 μm) of the region of interest were cut on ice-cold PBS with a vibratome (Leica VT1000, Leica, Germany) according to plates 21–23 (approximately from antero-posterior (AP) 1.18 to AP 0.98 mm from bregma) of the Paxinos and Franklin (2001) mouse brain atlas. Sections were then processed under the diaminobenzidine (DAB) technique to quantify neurons positive to the phospho (44/42)-extracellular signal-regulated kinases (pERK) as a marker of neuronal activation. After three rinses in PBS, sections were first incubated for 30 min in 1% H_2_O_2_, and then for 1 h in 3% bovine serum albumin (BSA) (Sigma-Aldrich, Milan, Italy). The incubation with the primary anti pERK antibody (Cell Signalling Technology, Beverly, MA, USA (1:350)) was conducted overnight at 4 °C. The following day, after rinsing in PBS, slices were incubated for 1 h with the biotinylated secondary antibody (1:800). After three rinses, slices were incubated in an avidin biotin peroxidase complex prepared according to the manufacturer’s suggestions (Vectastain ABC kit, Vector Laboratories, Burlingame, CA, USA) and a 3–3′-diaminobenzidine solution (10 mg/ml) was added until development of brown staining. Finally, sections were mounted onto glass slides coated with gelatine in Eukitt mounting medium for microscope visualization. Standard control experiments were performed by omission of the primary or secondary antibody and yielded no cellular labelling (data not shown). Images were obtained with an epifluorescence microscope (Axio Scope A1, Zeiss, Germany) connected to a digital camera (1.4 MPixels, Infinity 3–1, Lumenera, Canada). Brain sections immune-stained for pERK were evaluated using a 10X objective lens to acquire two images representing the whole left and right AcbSh. Then, the total number of pERK positive neurons was counted by using the manual particle counting option of ImageJ software (U.S. National Institutes of Health, Bethesda, MD, USA). Analysis was performed in a blinded manner. Since no significant differences in the counts of pERK-positive neurons were found amongst the two coronal sections of the AcbSh from the same mouse, values obtained from these sections were averaged.

### Statistical analysis

To determine statistically significant differences between pre-conditioning values of the experimental groups, one-way analysis of variance (ANOVA) was applied. To determine the effects of pre-treatment (vehicle or DF) and treatment (vehicle or ethanol or morphine) as well as of their interaction on acquisition of CPP, data were analysed by three-way ANOVAs with pre-treatment and treatment as independent factors (between subjects), and with pre-conditioning and post-conditioning values as a within-subjects factor (repeated measures). To determine the statistically significant effects of DF on CPP expression, three-way ANOVAs, with preference times (pre- and post-conditioning) as dependent factors, and with conditioning treatment (Veh or EtOH or MOR) and post-conditioning test treatment (Veh or DF) as independent factors, as well as their interactions, were conducted. All statistical analyses were carried out (StatSoft, v. 8.0, StatSoft Inc., Tulsa (OK), USA) using data from the experimental groups depicted in each figure. Newman-Keuls post-hoc analyses also between pre- and post-conditioning times within each conditioning group were undertaken if significant effects were found (*p* < 0.05).

pERK-positive neurons/area following each treatment were expressed as the average number of pERK-positive neurons/area of each experimental group and indicated as pERK-positive neurons/area (pERK expression). These values were used for statistical analyses by two-way ANOVAs with pERK-positive neurons/area as dependent variables and with pre-treatment (vehicle or *WSE* or DF) and treatment (vehicle or ethanol or morphine) as independent variables. All statistical analyses were carried out using data from the experimental groups depicted in each figure. Newman-Keuls post-hoc analyses were undertaken if significant effects were found (*p* < 0.05).

## Results

### Effects of DF on acquisition and expression of ethanol-induced CPP

**Fig. 1:**
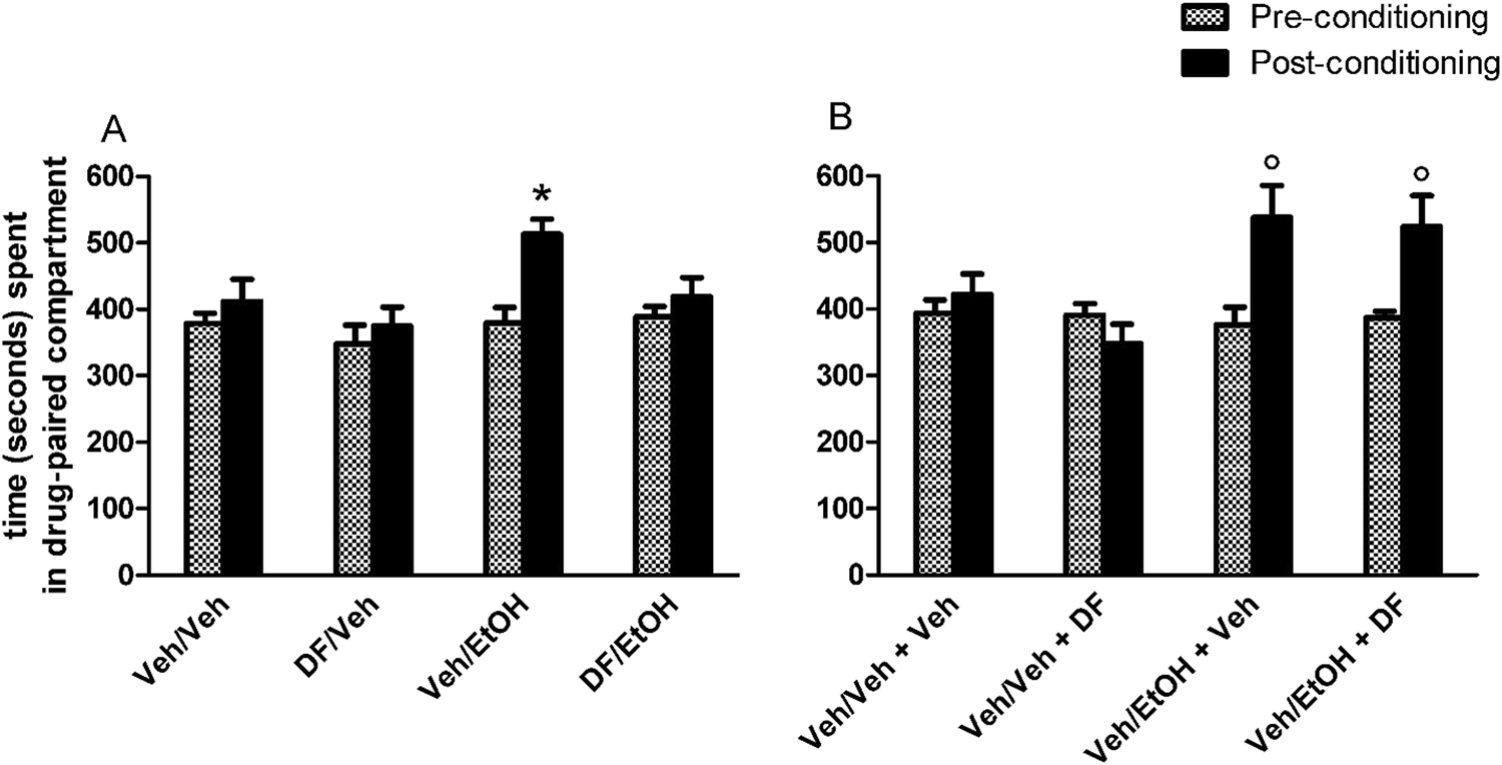
Effects of DF (2 mg/kg i.p.) on ethanol-induced CPP acquisition (**A**) and expression (**B**). Histograms represent the time (seconds/900) spent in the drug-paired compartment before and after conditioning expressed as mean + SEM. *indicates a significant difference (*p* < 0.05) vs pre-conditioning, same experimental group, in Fig. 1A; °indicates a significant difference (*p* < 0.05) vs pre-conditioning, same experimental groups, in Fig. 1B

Figure 1A shows the effects of pre-treatment with vehicle (Veh) or DF (2 mg/kg) 30 min before the administration of vehicle (Veh) or ethanol (EtOH) and exposure to the associated compartment for 30 min. One-way ANOVA revealed that pre-conditioning preference times did not significantly differ between experimental groups (*p* > 0.05). Repeated measures three-way ANOVA with preference times (pre- and post-conditioning) as dependent factors, and with pre-treatment (Veh or DF) and treatment (Veh or EtOH) as independent factors, revealed significant effects of time [*F*_(1,52)_ = 9.40, *p* < 0.005], pre-treatment [*F*_(1,52)_ = 4.39, *p* < 0.05] and treatment [*F*_(1,52)_ = 6.58, *p* < 0.05] confirming that EtOH stimulates a significant preference shift (Porru et al. 2020; Spina et al. 2015), indicating that DF on its own is devoid of conditioning properties (Maccioni et al. 2021) and suggesting that it may significantly prevent the acquisition of CPP induced by EtOH (*p* < 0.05). Figure 1B shows the effects of treatment with vehicle (Veh) or DF (2 mg/kg) 30 min before the exposure to the two compartments for the post-conditioning test of mice conditioned with (pre-treatment/treatment) Veh/Veh and Veh/EtOH. One-way ANOVA revealed that pre-conditioning preference times did not significantly differ between experimental groups (*p* > 0.05). Repeated measures three-way ANOVA with preference times (pre- and post-conditioning) as dependent factors, and with conditioning treatment (Veh or EtOH) and post-conditioning test treatment (Veh or DF) as independent factors, revealed significant effects of time [*F*_(1,35)_ = 9.59, *p* < 0.005] and conditioning-treatment [*F*_(1, 35)_ = 7.96, *p* < 0.05], and a significant time by conditioning-treatment interaction [*F*_(1,35)_ = 11.69, *p* < 0.005] but not a significant effect of post-conditioning test treatment [*F*_(1,35)_ = 0.71, *p* > 0.05] confirming that EtOH stimulates a significant preference shift (*p* < 0.05) and that DF fails to prevent this effect; in fact, EtOH-conditioned and DF-treated (30 min before the post-conditioning test) mice had a significant shift from 387 ± 10 to 524 ± 47 s/900 (*p* < 0.05), underlying that DF fails to affect the expression of EtOH-induced CPP.

### Effects of DF on acquisition and expression of morphine-induced CPP

**Fig. 2:**
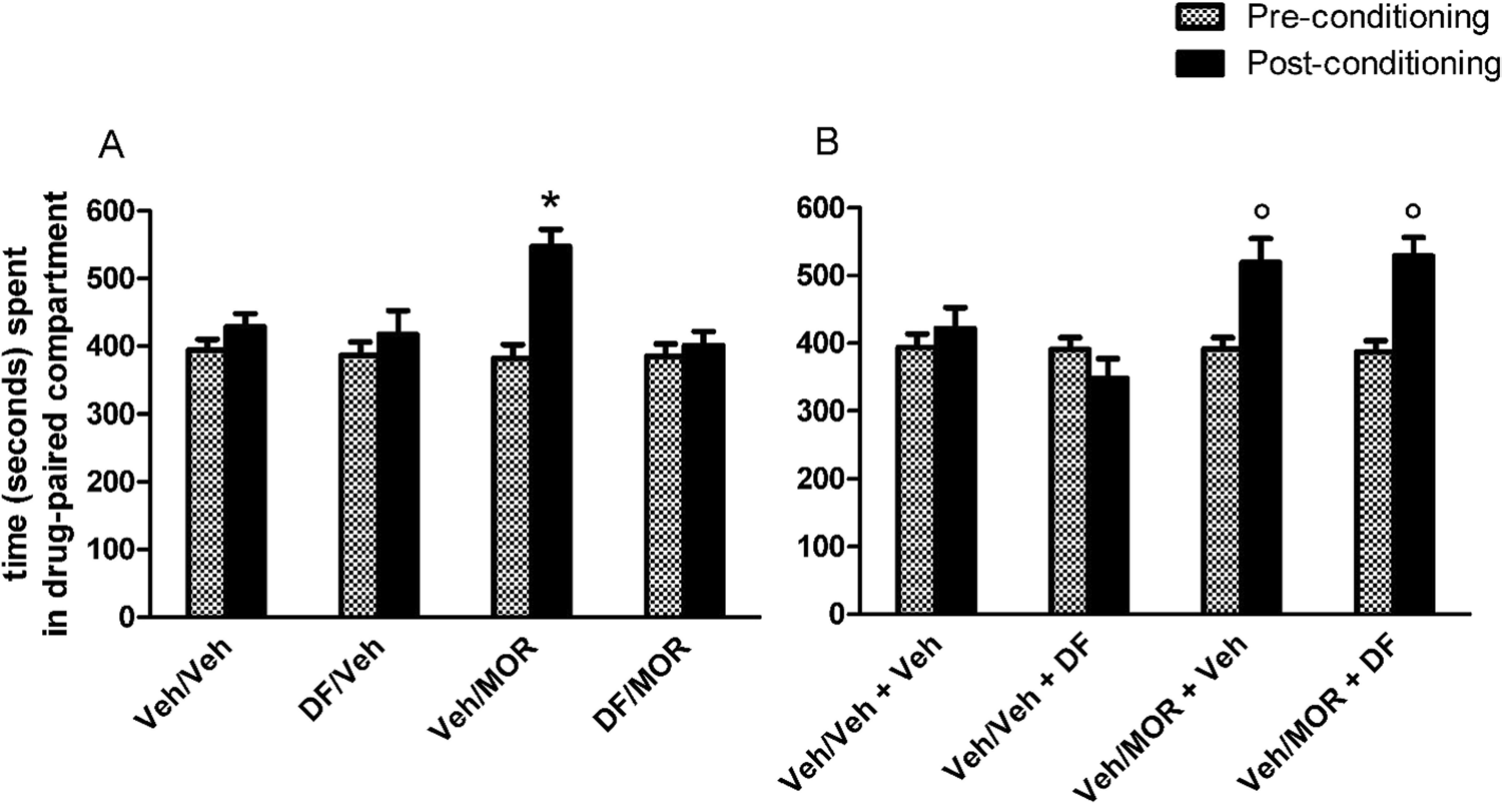
Effects of DF (2 mg/kg i.p.) on morphine-induced CPP acquisition (**A**) and expression (**B**). Histograms represent the time (seconds/900) spent in the drug-paired compartment before and after conditioning expressed as mean + SEM. *indicates a significant difference (*p* < 0.05) vs all groups in Fig. 2A; °indicates a significant difference (*p* < 0.05) vs the Veh/Veh + Veh and Veh/Veh + DF groups in Fig. 2B

Figure 2A shows the effects of pre-treatment with vehicle (Veh) or DF (2 mg/kg), 30 min before the administration of vehicle (Veh) or morphine (MOR) and exposure to the associated compartment for 30 min. One-way ANOVA revealed that pre-conditioning preference times did not significantly differ between experimental groups (*p* > 0.05). Repeated measures three-way ANOVA with preference times (pre- and post-conditioning) as dependent factors, and with pre-treatment (Veh or DF) and treatment (Veh or MOR) as independent factors, revealed significant effects of time [*F*_(1,40)_ = 14.02, *p* < 0.005] and pre-treatment [*F*_(1,40)_ = 7.05, *p* < 0.05] and significant time by pre-treatment [*F*_(1,40)_ = 5.34, *p* < 0.05] and time by pre-treatment by treatment [*F*_(1, 40)_ = 4.85, *p* < 0.05] interactions. Post-hoc analysis using the Newman-Keuls test confirmed that morphine stimulates a significant preference shift from 382 ± 20 to 547 ± 26 s/900 (*p* < 0.05) (Ruiu et al. 2013) and that DF is devoid of conditioning properties (Maccioni et al. 2021) and showed that DF significantly prevents the acquisition of CPP induced by morphine (*p* < 0.05). Figure 2B shows the effects of treatment with vehicle (Veh) or DF 30 min before exposure to the two compartments for the post-conditioning test of mice conditioned with Veh/Veh and Veh/MOR (pre-treatment/treatment). One-way ANOVA revealed that pre-conditioning preference times did not significantly differ between experimental groups (*p* > 0.05). Repeated measures three-way ANOVA with preference times (pre- and post-conditioning) as dependent factors, and with conditioning (Veh or MOR) and post-conditioning test treatments (Veh or DF) as independent factors, revealed significant effects of time [*F*_(1,36)_ = 11.24, *p* < 0.005] and conditioning-treatment [*F*_(1, 36)_ = 15.73, *p* < 0.005] and a significant time by conditioning-treatment interaction [*F*_(1,36)_ = 14.01, *p* < 0.001] but not a significant effect of post-conditioning test treatment [*F*_(1,36)_ = 1.10, *p* > 0.05] confirming that morphine stimulates a significant preference shift from 392 ± 16 to 519 ± 35 s/900 (*p* < 0.05) and that the post-conditioning test treatment with DF fails to prevent this effect; in fact, morphine-conditioned and DF-treated (30 min before the post-conditioning test) mice had a significant shift from 391 ± 17 to 525 ± 28 s/900 (*p* < 0.05), underlying that DF fails to affect the expression of morphine-induced CPP.

### Effects of WSE on ethanol- and morphine-induced ERK phosphorylation in AcbSh

**Fig. 3:**
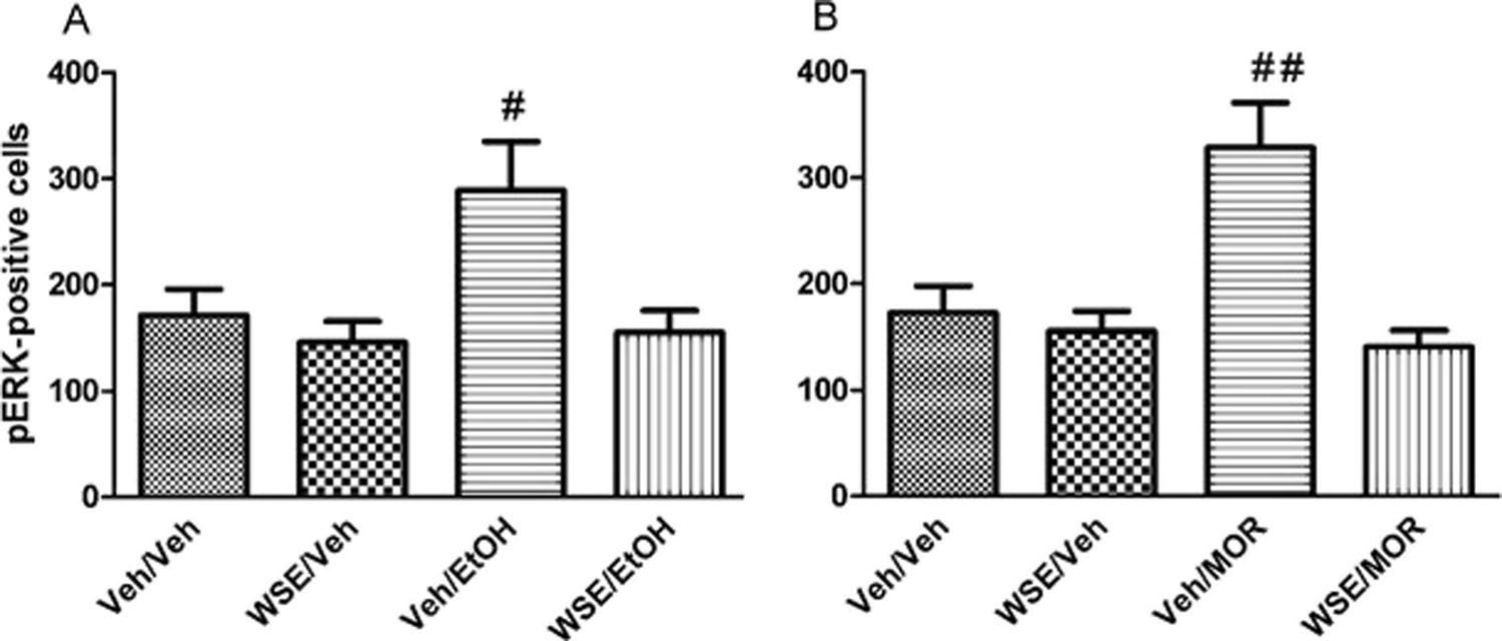
Effects of acute administration of *WSE* (50 mg/kg i.p.) on the expression of ethanol (2 g/kg i.p.)- (**A**) and morphine (5 mg/kg i.p.)- (**B**) elicited pERK-positive neurons in the AcbSh of adult male CD1 mice. Data are shown as mean + SEM of pERK-positive neurons/area. ^**#**^indicates a significant difference (*p* < 0.05) vs pre-conditioning, same experimental group in Fig. 3A; ^##^indicates a significant difference (*p* < 0.05) vs all groups in Fig. 3B

Figure 3A shows the effects of pre-treatment with vehicle (Veh) or *WSE* (50 mg/kg) 30 min before the administration of vehicle (Veh) or ethanol (EtOH, 2 g/kg) (treatment) on the number of pERK-positive neurons (pERK expression) in the AcbSh. Two-way ANOVA with pre-treatment and treatment as categorical variables and pERK-positive cells counts as dependent variable revealed significant effects of pre-treatment [*F*_(1, 24)_ = 7.86; *p* < 0.05) and treatment [*F*_(1, 24)_ = 5.03; *p* < 0.05) confirming that ethanol increases the number of pERK-positive neurons (Ibba et al. 2009; Porru et al. 2020) and suggesting that pre-treatment with *WSE* may significantly prevent ethanol-induced ERK phosphorylation (*p* < 0.05). Figure 3B shows the effects of pre-treatment with vehicle (Veh) or *WSE* (50 mg/kg) 30 min before the administration of vehicle (Veh) or morphine (MOR, 5 mg/kg) (treatment) on the number of pERK-positive neurons in the AcbSh. Two-way ANOVA with pre-treatment and treatment as categorical variables and pERK-positive cells counts as dependent variable revealed significant effects of pre-treatment [*F*_(1,30)_ = 15.04; *p* < 0.005) and treatment [*F*_(1,30)_ = 7.16; *p* < 0.05] and a significant pre-treatment by treatment interaction [*F*_(1,30)_ = 10.40; *p* < 0.005]. Post-hoc analysis using the Newman-Keuls test confirmed that morphine increases the number of pERK-positive neurons in the AcbSh (Rosas et al. 2016) and showed that pre-treatment with *WSE* significantly prevents this effect (*p* < 0.05). Representative images of these effects are shown in Fig. 5.

### Effects of DF on ethanol- and morphine-induced ERK phosphorylation in AcbSh

**Fig. 4:**
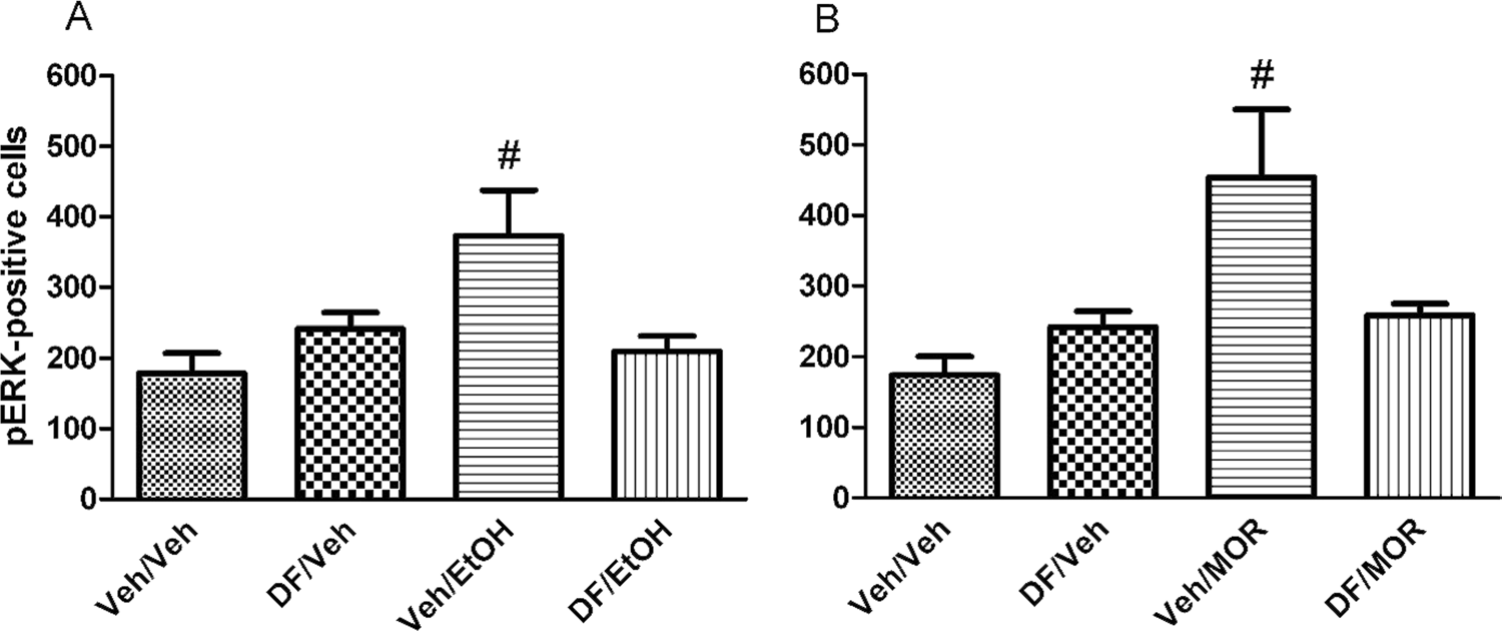
Effects of acute administration of DF (2 mg/kg i.p.) on the expression of ethanol (2 g/kg i.p.)- (**A**) and morphine (5 mg/kg i.p.)- (**B**) elicited pERK-positive neurons in the AcbSh of adult male CD1 mice. Data are shown as mean + SEM of pERK-positive neurons/area. ^**#**^indicates a significant difference (*p* < 0.05) vs all groups

**Fig. 5:**
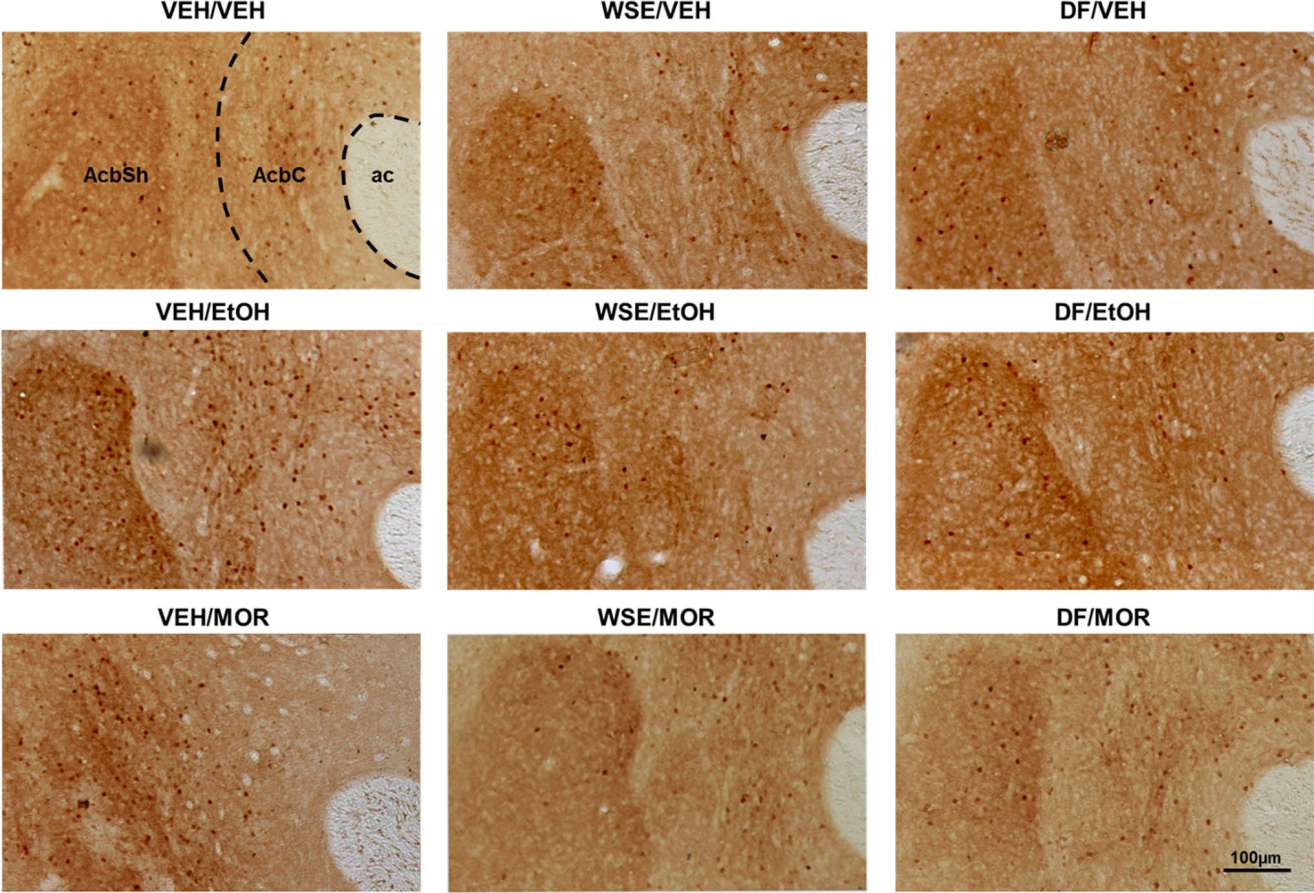
Low (10X) magnification demonstrative images of the effects of the acute administration of *WSE* and DF on ethanol- and morphine-elicited pERK-immunostaining in the AcbSh from mice representative of pre-treatment/treatment groups shown in Figs. 3 and 4. Abbreviations: *AcbC*, nucleus accumbens core; *AcbSh*, nucleus accumbens shell; *ac*, anterior commissure

Figure 4A shows the effects of pre-treatment with Veh or DF (2 mg/kg) 30 min before the administration of vehicle (Veh) or ethanol (EtOH, 2 g/kg) (treatment) on the number of pERK-positive neurons (pERK expression) in the AcbSh. Two-way ANOVA with pre-treatment and treatment as categorical variables and pERK-positive cells counts as dependent variable revealed significant effects of treatment [*F*_(1,27)_ = 4,95; *p* < 0.05] and a significant pre-treatment by treatment [*F*_(1,27)_ = 9,58; *p* < 0.005] interaction. Post-hoc analysis using the Newman-Keuls test confirmed that EtOH increases the number of AcbSh pERK-positive neurons (Ibba et al. 2009; Porru et al. 2020) and showed that pre-treatment with DF prevents this effect (*p* < 0.05). Figure 4B shows the effects of pre-treatment with vehicle (Veh) or DF (2 mg/kg) 30 min before the administration of vehicle (Veh) or morphine (MOR, 5 mg/kg) on the number of pERK-positive neurons in the AcbSh. Two-way ANOVA with pre-treatment and treatment as categorical variables and positive cells counts as dependent variable revealed significant effects of treatment [*F*_(1,28)_ = 7.97; *p* < 0.05] and a significant pre-treatment by treatment [*F*_(1,28)_ = 6.62; *p* < 0.05] interaction. Post-hoc analysis using the Newman-Keuls test confirmed that morphine increases the number of AcbSh pERK-positive neurons (Rosas et al. 2016) and showed that pre-treatment with DF significantly prevents this effect (*p* < 0.05). Representative images of these effects are shown in Fig. 5.

## Discussion

Previous studies have shown that *WSE* prevents both ethanol (Spina et al. 2015)- and morphine (Ruiu et al. 2013)-elicited acquisition of CPP as well as both ethanol- and morphine-elicited CPP expression, i.e. the ability of environmental stimuli, conditioned to both ethanol (Spina et al. 2015) and morphine (Ruiu et al. 2013), to determine a positive side-preference shift (CPP expression) at the post-conditioning test.

In fact, failure of classical pharmacological approaches (Lu et al. 2006) to treat drug addiction in humans has triggered the scientific interest on the application of phytomedicine and natural remedies for the treatment of drug addiction. In particular, the efficacy of *WSE* in pre-clinical models of drug addiction is strongly supported by over a decade of research (Gupta and Rana 2008; Kasture et al. 2009; Kulkarni and Ninan 1997; Peana et al. 2014; Ruiu et al. 2013; Spina et al. 2015; Bassareo et al. 2019). Thus, as a follow-up of previous studies from our laboratory, the present investigation was aimed at (**i**) characterizing further the potentially beneficial application of *WSE* to counteract the ability of acutely administered ethanol (Ibba et al. 2009; Porru et al. 2020, 2021; Rosas et al. 2017) and morphine (Rosas et al. 2016) to increase ERKs phosphorylation in the AcbSh and (**ii**) establishing whether *WSE*’s active compound, docosanyl ferulate (Maccioni et al. 2021; Sonar et al. 2019), may be responsible for such effects as well as for *WSE*’s effects on acquisition and expression of ethanol- and morphine-elicited CPP. The results of the present behavioural experiments confirm that ethanol (Spina et al. 2015) and morphine (Ruiu et al. 2013) elicit a significant CPP. The present results also reveal that DF, at the dose of 2 mg/kg, fully effective in exerting anxiolytic properties, without showing sedative, amnesic, motor impairing and motivational effects (Maccioni et al. 2021), significantly prevents the acquisition (Figs. 1A and 2A) but not the expression (Figs. 1B and 2B) of ethanol- and morphine-elicited CPP. Moreover, these data appear in partial agreement with our previous reports on the effects of *WSE* on acquisition and expression of ethanol- and morphine-elicited CPP, suggesting that the GABA_A_R-mimetic component, represented by DF, is critical for *WSE*’s ability to affect the acquisition but not the expression of CPP elicited by ethanol and morphine. This interpretation is supported by the observation that AcbSh DA has been reported to be critical for the acquisition, but not the expression, of morphine-elicited CPP (Fenu et al. 2006) and appears overall in agreement with the role played by mesolimbic DA in the associative learning (Di Chiara 1998; Di Chiara and Bassareo 2007) at the basis of CPP acquisition (Di Chiara et al. 2004). Accordingly, via a GABA_A_R-mediated mechanism, *WSE* was reported to significantly suppress the stimulatory actions of both ethanol and morphine on the neuronal firing of ventral tegmental area DA neurons (Bassareo et al. 2019) and to prevent ethanol- and morphine-mediated increases of AcbSh DA release (Bassareo et al. 2019).

The complex relationship between GABA_A_R modulators and the reinforcing properties of both ethanol and morphine has been addressed, although with no conclusive results, in the literature. In particular, studies investigating the interactions between GABA_A_R modulators and the reinforcing properties of ethanol showed that GABA_A_R ligands, both agonists (Hodge et al. 1995) and antagonists (Hodge et al. 1995; June et al. 1998) reduce ethanol self-administration and that GABA_A_R antagonists increase ethanol-induced CPP and conditioned taste aversion in mice (Chester and Cunningham 1999). Moreover, in addition to such uncertainty on the role of GABA_A_R on the reinforcing properties of ethanol and morphine, these results also suggest that rather than being related to the actions of ethanol and morphine, the involvement of GABA_A_R may be related to their critical role in the learning process at the basis of the acquisition of the conditioned response. Furthermore, the present behavioural findings also indicate that other components of *WSE*, besides DF, may be responsible for *WSE*’s ability to affect the expression of drug-induced CPP. This conclusion is fully compatible, in a complementary perspective, with the observation that distinct neural processes and anatomical structures may differentially underlie distinct phases of drug-elicited place conditioning (Bardo 1998; Tzschentke 2007).

The results of the present study also confirm that both ethanol (Porru et al. 2020, 2021; Rosas et al. 2017) and morphine (Rosas et al. 2016; Valjent et al. 2004) activate ERKs phosphorylation in the AcbSh of CD1 mice and show for the first time that both *WSE* (50 mg/kg) and DF (2 mg/kg) are able to prevent these increases. The acute effects of *WSE* and DF in the prevention of either ethanol- or morphine-induced ERKs phosphorylation in the AcbSh was assessed, in distinct cohorts of animals, to better define the molecular mechanisms leading to *WSE*’s and DF’s prevention of the acquisition of ethanol- or morphine-elicited CPP. Thus, whilst we acknowledge that this evidence is indirect, since obtained from mice that had no conditioning, our suggestion that *WSE* and DF may contrast the acquisition of ethanol- or morphine-elicited CPP by averting ERKs phosphorylation in the AcbSh is also supported by the critical role of pERK in associative learning (Gerdjikov et al. 2004; Marotta et al. 2014; Salzmann et al. 2003). Moreover, these findings appear overall in agreement with the observation that activated ERKs play a critical role in the conditioned approach behaviour elicited by addictive drugs as assessed in the place conditioning paradigm (Gerdjikov et al. 2004; Lu et al. 2006; Rosas et al. 2018; Salzmann et al. 2003; Spina et al. 2010; Valjent et al. 2000, 2001) but also with the ability of *WSE*, via a GABA_A_R-mediated mechanism, to control ethanol- and morphine-stimulated AcbSh DA transmission (Bassareo et al. 2019) as well as with the role of DA in ethanol (Ibba et al. 2009)- and morphine (Rosas et al. 2016)-elicited ERK phosphorylation in the AcbSh. However, whilst all this reasoning applies coherently to the recognition of the mesolimbic dopaminergic system in place conditioning (Di Chiara et al. 2004; Tzschentke 2007), we also acknowledge that other brain areas, such as the hippocampus (Bagherpasand et al. 2019; Zhang et al. 2016) as well as pERK expression therein (Bagherpasand et al. 2019; Zhang et al. 2016), may be critically responsible for the acquisition of place conditioning. The relationship between GABA_A_R and ERKs phosphorylation can also be interpreted in light of the observation that a putative phosphorylation site for ERKs was found in almost all known alpha subunits of the GABA_A_R, including the ubiquitously expressed alpha1 subunit (Bell-Horner et al. 2006). Interestingly, this study demonstrated that this site is functional and that pERK acts as a negative GABA_A_R modulator as its inhibition, through pharmacological inhibition of MEK, results in an amplification of GABA_A_R currents in HEK293 cells (Bell-Horner et al. 2006). Hence, the relationship between pERK and GABA_A_R might be bidirectional, as GABA_A_R agonists lead to a decrease in pERK expression and reduction of pERK expression by MEK inhibition, in turn, induces an increase of GABA_A_R-mediated currents.

Overall, the previous (Ruiu et al. 2013; Spina et al. 2015) and present data on *WSE* support the view that selective products of phytomedicine and natural remedies may be useful strategies for the treatment of brain disorders as well as for further understanding the underpinning subcellular mechanisms. The relevance of these findings comes not only from this observation but also because DF’s data contribute significantly to characterize the relationship between *WSE*, pERK, two distinct phases of the place conditioning paradigm and GABA_A_R pointing to DF as a potential pharmacological agent for the management of drug addiction. In fact, DF already resulted as a promising molecule in its first behavioural characterization, which showed an anxiolytic activity comparable to the GABA_A_R positive modulator, diazepam (Maccioni et al. 2021; Nutt and Blier 2016), however combined with the lack of undesired motor and mnemonic effects, of addictive potential as well as of the diazepam’s property to potentiate ethanol’s depressant central effects (Maccioni et al. 2021).

In conclusion, these results may support the suggestion of the suitability of both *WSE* and DF as strategies for the management of distinct phases of drug addiction, the establishment of associative memories (modelled by the CPP acquisition) and the triggering of drug-seeking by contextual conditioned stimuli (modelled by the CPP expression). This suggestion is further supported on one hand by the observation that both *Withania somnifera* (Dar et al. 2015) and DF (Maccioni et al. 2021) present a robust anxiolytic profile and, on the other hand, by the observation that anxiety disorders and drug addiction may co-occur at high rates (Smith and Book 2008). Hence, the anxiolytic profile and the ability to prevent ethanol- and morphine-elicited CPP may truly be useful in the development of an efficient therapeutical strategy, especially considering the lack of undesired effects. Additional behavioural and biochemical studies will have to be performed to characterize further their suitability for the treatment of drug addiction.
